# Implications of the foliar phytochemical diversity of the avocado crop *Persea americana* cv. Hass in its susceptibility to pests and pathogens

**DOI:** 10.7717/peerj.11796

**Published:** 2021-07-20

**Authors:** Francisco J. Espinosa-García, Yolanda M. García-Rodríguez, Angel E. Bravo-Monzón, Ernesto V. Vega-Peña, Guillermo Delgado-Lamas

**Affiliations:** 1 Instituto de Investigaciones en Ecosistemas y Sustentabilidad, Universidad Nacional Autónoma de México, Morelia, Michoacán, México; 2 Escuela Nacional de Estudios Superiores Mérida, Universidad Nacional Autónoma de México, Mérida, Yucatán, México; 3 Instituto de Química, Universidad Nacional Autónoma de México, Ciudad de México, Mexico

**Keywords:** Greater diversity-better defense hypothesis, Avocado pests and pathogens, *Copturus aguacatae*, Phytochemical diversity, *Sphaceloma perseae*, Secondary metabolite chemotype diversity, *Colletotrichum gloeosporioides*, *Oligonychus* spp.

## Abstract

Phytochemical diversity (PD) can be considered as a defensive trait; it can operate through single plant secondary metabolites or usually as complex mixtures of them. We tested the more diversity-better defense hypothesis correlating the leaf plant secondary metabolites (PSMs) with the incidence of plant enemies on Hass avocado trees. We expected a negative correlation between the occurrence of plant enemies and PD metrics. Also, as intraspecific PSMs polymorphisms in plant populations are common, we studied the incidence of plant enemies on Hass avocado trees representing chemical variants (chemotypes). We expected a differential incidence of plant enemies among trees grouped by their mono and sesquiterpene + phenylpropanoid chemotypes. We analyzed foliar hexane extracts from 236 trees in 17 orchards by gas chromatography and for the incidence of red mite, thrips, whitefly, avocado branch borer, fruit rot, scab, and peduncle collar blight. The predicted negative correlation between the plant enemies’ incidence and the phytochemical metrics did not occur. To determine the relationship between enemy incidence and chemotypes we grouped the trees by cluster analysis using a matrix of PSMs in each tree. Most trees were grouped under four out of 23 chemotypes. Branch borers attacked trees of low-frequency chemotypes more frequently than trees with common chemotypes. The incidence of five plant enemies was different among the predominant chemotypes. The hypothesis of more diversity-better defense was not supported by the correlations between the phytochemical diversity and the incidence of pests and pathogens in Hass avocado orchards. Based on our results, we hypothesize that phytochemical diversity function as a defensive trait relies more on differentiation among individuals in a population than on the sole increase of chemical diversity. Also, the differential incidence of pests and pathogens on trees classified by their foliar chemotypes implies that these susceptibility or resistance markers represent potential useful tools for Hass avocado orchard pest management.

## Introduction

Dozens or even hundreds of pest and pathogen species attack plants; one of the most important mechanisms of resistance is chemical defense (Linhart, 1991; Paul, Hatcher & Taylor, 2000; Stam et al., 2014). Plant secondary metabolites (PSMs), with ca. 200,000 isolated and described (Kessler & Kalske, 2018), constitute the chemical defense of plants. PSMs are present in all plant species in mixtures that vary among plant tissues, individuals, and populations (Langenheim, 1994; Gershenzon et al., 2012). This phytochemical variability may be constitutive (genetically determined without external stimuli), induced by plant enemies or mutualists, phenology, physical environmental factors (e.g., water, light, nutrients, temperature), or occur during the development of individuals or plant organs (Lämke & Unsicker, 2018; Kessler & Kalske, 2018). Furthermore, such phytochemical variability develops in space and time resembling dynamic mosaics of chemical mixtures that plant enemies must face. Thus, phytochemical diversity should be considered as a defensive trait. Phytochemical diversity may operate through the effect of the sum of individual compounds or particular mixtures of PSMs effective against one or several plant enemies. Thus, to understand the function of phytochemical diversity, the full content of PSMs in a plant should be studied in relationship with its enemies. The variation of individual phenotypes is thought to be relevant for the persistence of plant populations because this diversity prevents the evolution of resistant strains or biotypes of plant enemies capable of annihilating plant populations (Pimentel & Bellotti, 1976; Gershenzon & Dudareva, 2007; Gershenzon et al., 2012; Pearse, Paul & Ode, 2018; Wetzel & Whitehead, 2020).

A fraction of the chemical profile of a phenotype, which may not be as variable as the whole set of displayed PSMs, is the relative composition of blends of biosynthetically related compounds when the relative composition is under tight genetic control. The polymorphisms in the relative composition of blends have been called compositional patterns, compositional types or chemotypes that can be used as markers of resistance or susceptibility to plant enemies (Langeheim & Stubblebine, 1983). That is the case for the relative composition of mono and sesquiterpenoids of conifers and some other trees and shrubs (Hanover, 1966; Von Rudloff, 1975; Langeheim & Stubblebine, 1983; Squillace, Powers Jr & Kossuth, 1985; Thompson et al., 2003; Wheeler, 2006; Bustos-Segura et al., 2017). Several studies on plant populations described polymorphisms on the blends of secondary metabolites (SM) that antagonize plant enemies (Squillace, Powers Jr & Kossuth, 1985; Leather, Watt & Forrest, 1987; Espinosa-García & Langenheim, 1991; Langenheim, 1994; Linhart et al., 2005). For example, the terpenoid chemotype of the leaves of some conifers is associated with the differential attack of mammals, mollusks, insects, or pathogenic fungi (Sturgeon & Mitton, 1986; Ennos & Swales, 1988; Espinosa-García & Langenheim, 1991; Snyder, 1992; Iason et al., 2012; Bustos-Segura & Foley, 2018). This differential susceptibility associated with chemotypes has also been observed in other plant species and their respective plant enemies (Langenheim, 1994; Kessler & Kalske, 2018).

In tropical rainforests, it is suggested that chemical differentiation among individuals allows them to survive in physical proximity to each other (Janzen, 1970; Connell, 1971; Langeheim & Stubblebine, 1983; Sánchez-Hidalgo, Martínez-Ramos & Espinosa-García, 1999; Comita et al., 2014). Nevertheless, cultivated plant species frequently exhibit a reduction in the amount of the genetic variation, as well as in the concentration and the diversity of their secondary metabolites (e.g., bitter and toxic compounds) when compared to their noncultivated counterparts (Lindig-Cisneros, 1997; Massei & Hartley, 2000; Meyer, DuVal & Jensen, 2012). The genetic homogenization of crops makes them vulnerable to the attack of devastating pests and pathogens (Chaudhary, 2013; Chen, Gols & Benrey, 2015; Whitehead, Turcotte & Poveda, 2017). This susceptibility can facilitate that diseases reach epidemic proportions in asexually propagated crops, such as potato or sugar cane, where genetic uniformity increases (Strange & Scott, 2005).

An early hypothesis explaining the defensive function of phytochemical diversity posits that the effectiveness of defense depends on diversity: the more phytochemical diversity, the more effective the defense (Berenbaum, 1985; Kubo & Hanke, 1985; Rhoades, 1985; Jones, Firn & Malcolm, 1991; Jones & Lawton, 1991; Richards et al., 2015). This hypothesis is paired with another postulating that high pathogen and herbivore pressure causes high phytochemical diversity (Jones & Lawton, 1991; Langenheim, 1994). Those hypotheses rely on the differential bioactivity of the PSMs on plant enemies and the ability of plant enemies to nullify PSMs differentially. The more diversity-better defense hypothesis supports the widely held observation that reduced genetic diversity in crops produces devastating pests and pathogens, and that increased genetic diversity prevents or retards the appearance of such organisms greatly (Pimentel & Bellotti, 1976; Gershenzon & Dudareva, 2007; Gershenzon et al., 2012; Tooker & Frank, 2012). Still, the amount and type of phytochemical diversity needed to prevent catastrophic losses in crops are not well defined. The prediction of more diversity-better defense can be fulfilled by the bioactivity of single compounds or by synergistic and additive effects in sets of compound mixtures in which each one affects plant enemies differentially (Langenheim, 1994; Gershenzon et al., 2012; Richards et al., 2016). However, the question of whether specific compounds or their mixtures need to be displayed in a plant population to coexist with its plant enemies remains open. Also, the possible existence of a lower and upper limit of phytochemical diversity in a plant population determining its viability against its enemies needs to be investigated.

Our aim in this study was to determine if the incidence of plant enemies is related to the phytochemical variation determined by individual metabolites or chemotypes in orchards of Hass avocado. We tested the more diversity-better defense hypothesis that predicts negative correlations between the incidence of plant enemies and phytochemical diversity. To test this prediction, we correlated the occurrence of plant enemies on Hass avocado trees with several phytochemical diversity metrics at the single tree and the orchard scales. We also wanted to determine if the type and frequency of chemotypes in the orchards are related to the incidence of plant enemies. We expected differential incidence of plant enemies on plant individuals associated with different chemotypes and their frequency in a population. Given that the Hass avocado is a recent cultivar that originated from one or a few individuals (Bost, Smith & Crane, 2013), and that plantings are clones, the expectation was to find very few chemotypes in the avocado orchards. However, the Hass cultivar shows significant phytochemical variability among individuals and orchards (García-Rodríguez et al., 2016). Also, considering that at least ten million avocado trees have been cloned in Mexico (ca. 100,000 hectares planted at the time of the study with 100–120 trees per hectare FIRA, 2007), and that the growers select and reproduce clonally branches with fruits of desirable characteristics, we supposed that the amount of variability in the Haas cultivar allowed the hypothesis testing.

## Study Area and Methods

### The Hass avocado avocado cultivar

*Persea americana* cultivar Hass, patented in 1935 by Rudolph Hass, is a hybrid of the Guatemalan (*P. americana* var. *guatemalensis*) and Mexican (*P. americana* var. *drymifolia*) botanical varieties (Fiedler, Bufler & Bangerth, 1998; Chen et al., 2009; Rendón-Anaya et al., 2019). At present, Hass avocado is the cultivar that occupies the largest planted surface in Michoacán, where it has replaced other varieties almost entirely (Sánchez-Colín et al., 2001). Around 168,000 hectares in the state of Michoacán, Mexico, are planted to Hass avocado cloned by grafting on stocks of Mexican avocado (SAGARPA-SIAP, 2019).

Numerous herbivores and pathogens of Hass avocado cause sanitary problems (Dann et al., 2013; Peña et al., 2013) that force the avocado growers to establish scheduled applications of pesticides in their orchards to prevent harvest losses. The attack of pests and pathogens on Hass avocado may be fostered by the reduced variation of the crop, which is produced by domestication and clonal propagation by grafting (Tooker & Frank, 2012).

*Persea americana* is rich in secondary metabolites with antagonistic activity against herbivores and pathogens (Sneh & Gross, 1981; Brune & Van lelyveld, 1982; Prusky et al., 1985; Hennessey, Knight & Schnell, 1995). The most studied secondary metabolites in *Persea* spp. are terpenoids and phenylpropanoids from the essential oils of leaves (Montes, Valenzuela & Wilkomirsky, 1981; Lavi et al., 1993a; Weyerstahl, Marschall & Scora, 1993; Scora & Ahmed, 1993; Sagrero-Nieves & Bartley, 1995), although other bioactive compounds are present in more tissues of the plant (Oelrichs et al., 1995; González-Coloma et al., 1996; Torres-Gurrola et al., 2016). Avocado trees with various leaf chemotypes of mono- and sesquiterpenes and phenylpropanoids have been detected in avocado orchards where one or two chemotypes may predominate (García-Rodríguez et al., 2016; Meléndez-González & Espinosa-García, 2018).

### Study area

Seventeen productive orchards (avg. 20 years old) were studied in the ‘avocado belt’ in Michoacán, which spans from the municipality of Tacámbaro to that of Los Reyes, over an altitudinal range of 600 to 2,260 m above sea level (Appendix S1). The orchards in the region receive six to 12 applications per year of insecticides, fungicides, foliar fertilizers, or hormones (FIRA, 2007); pesticides represent 31.5 to 50% of the production costs, and each manager decides the type, number, and time of application (Franco Sánchez et al., 2018). The orchards’ owners or managers gave us oral permission to collect avocado leaves and data on avocado pests and pathogens in their properties.

### Records of pests and pathogens

The fieldwork was conducted from July to November of 1999 and from January to June of 2000. Records were made of the incidences of red mite (*Oligonychus yothersi*, and other species from the same genus), avocado branch borer (*Copturus aguacatae*), whitefly (*Paraleyrodes perseae, Trialeurodes spp*.), thrips (*Liothrips perseae*, *Heliothrips haemorrhoidalis*, and other species), fruit rot or anthracnose (*Colletotrichum gloeosporioides*), scab (*Sphaceloma perseae*), and peduncle collar blight, PCB (caused by a complex of several bacteria and, possibly, fungi). For each tree, the records of incidence for pests and pathogens were made as follows: for red mite, whitefly, and thrips, the number of animals in the terminal leaves of four branches, one in each cardinal point; for branch borer, the number of damaged branches in four canopy zones (damaged branches show insect-bored entrance holes that exude a whitish liquid), each one coincident with one of the four cardinal points; for fruit rot (anthracnose) and scab, the number of fruits showing typical symptoms of each disease; for PCB, the number of fallen fruits under the canopy of the tree. Fruits attacked by anthracnose show sunken necrotic lesions, circular and mushy; mature leaves show brown spots, and tender leaves, twigs, and flowers can also be attacked, drying out resembling frost damage. The shell of fruits attacked by scab show corky (cracked and scraped) brown lesions of irregular shape. PCB causes an incision or ring close to the union of the branchlet and the peduncle of the fruit that breaks down producing an abundant immature fruit fall (Equihua, Estrada & González, 2007; Paleo et al., 2015; Vidales, 1993; Vidales, 1996; Salgado, 1993).

### Sample processing

In each of the 17 orchards, 15 trees were randomly selected (excluding trees located on the edge of dirt roads). Four mature leaves with their petioles (one in each cardinal point: N, S, E, and W) were taken from each tree for chemical analysis. Accidentally, we lost 19 samples for chemical analysis, leaving a final sample size of 236 trees (Appendix S1). Leaves collected from each tree were cut along the midrib after discarding the petiole, and each half was weighed to obtain fresh weight. One half was macerated in amber glass flasks with 70 mL of hexane plus 1 mg of tetradecane added as an internal standard. This process was conducted in each orchard after leaf sampling was completed. The other leaf halves were oven-dried at 50 °C to obtain the dry weight, which was used to calculate the water content of the fresh leaves, and then, to calculate the dry weight of the macerated halves. On average, 1.2 g of fresh leaves were macerated. The 70 mL of hexane extract was filtered and dried with magnesium sulfate and pooled with successive dry hexane extracts obtained by grinding the macerated leaves in a mortar with fresh hexane and neutralized and washed sand. Successive extractions were performed until the hexane extract was clear (up to four extractions). The pooled hexane extracts were evaporated under a gentle stream of nitrogen in 50 mL glass centrifuge tubes (washing the extract remnants from the tube walls with clean hexane) to 10 mL that were transferred to a 15 mL glass centrifuge tube to continue the evaporation process and obtain 0.2 mL to inject 2 μL into the chromatograph (see ‘Analysis and quantification of the chromatographic peaks obtained by gas chromatography coupled to flame ionization detector (GC-FID)’). The remainder was transferred to a one mL vial, which was filled with the hexane used to wash the walls of the centrifuge tube. Subsequently, a small amount of pyrogallol (Sigma-Aldrich) was added as an antioxidant (Torres-Gurrola, Montes-Hernández & Espinosa-García, 2009). The vials were stored at −20 °C until further analysis.

### Analysis and quantification of the chromatographic peaks obtained by gas chromatography coupled to flame ionization detector (GC-FID)

Two μL were injected into a Perkin-Elmer 8410 gas chromatograph coupled to a flame ionization detector, equipped with a 30 m Alltech Econo-Cap™ EC™-5 C column (5% Phenyl 95% dimethylpolysiloxane) with 0.25 mm ID and 25 μm film thickness, and a P-E 1020 integrator. Helium was the carrier gas at 100 mL min^−1^. The injection mode was split (50:1) with the injector at 225 °C; the initial oven temperature was 50 °C, increased at 15 °C min^−1^ until 225 °C, and then maintained for 15 min. After this, a ramp of 10 °C min^−1^ was applied until 300 °C and then sustained for 8 min. The FID detector was 350 °C.

Once one chromatogram per tree was obtained, the peaks’ retention times were estimated relative to the tetradecane internal standard to control the variability among runs of the retention times of the peaks. The concentration of each peak was calculated as micrograms g^−1^ of leaf dry weight relative to tetradecane.

#### Peak identification by gas chromatography coupled to mass spectrometry (GC-MS)

Twenty hexane extracts, previously analyzed with GC-FID, were analyzed by GC-MS. Each one mL hexane extract was concentrated until 0.2 mL under a soft nitrogen stream. We prevented monoterpenoid loss avoiding the evaporation to dryness taking advantage of the cooling effect produced by the evaporation of hexane. From each extract, 1 μL was injected into an Agilent 6890 Gas Chromatograph coupled to an Agilent 5973N selective mass detector equipped with an HP-5MS (5%Phenyl 95% dimethylpolysiloxane) capillary column (30 m × 0.25 mm with 0.25 mm film thickness). Helium was the carrier gas at 7.67 psi with a 1.0 mL min^−1^ constant flow. The front inlet was 250 °C in a split ratio of 60.3:1. The initial oven temperature was 50 °C, that increased to 200 °C at 20 °C min^-1^, and then to 280 °C at 15 °C min^-1^, and finally, increased to 300 °C at a 20 °C min^-1^. The mass spectrometer was in electrical ionization mode (EI), with flow one mL min^-1^, 70 eV ionization voltage, the interface temperature at 280 °C, and a scan range of 35 –500 m/z.

The identification of the components was made by comparing their Kovats retention indices with those reported in the literature. The calculation of the Kovats indices was made based on a linear interpolation of the retention time of homologous series of n-alkanes (C8 –C20; C21-C40) under the same GC-MS operating conditions and column used to obtain the peaks. Also, the components were identified by comparing the mass spectra of each constituent with those stored in the Wiley/NIST 02.L database and with mass spectra from the literature (Linstrom & Mallard, 2001; Adams, 2007). The criterion used for including a compound in the data set was that the signal of its peak was equal or greater than 10X the chromatogram baseline noise level.

Persin and 2-[(8Z,11Z)-heptadeca-8,11-dienyl]-furan were identified by mass fragmentation patterns comparison from the literature (Oelrichs et al., 1995; Rodriguez-Saona et al., 1998; Meléndez-González & Espinosa-García, 2018). The peaks of four contaminant compounds (phthalates and silanes) and pyrogallol, were eliminated leaving 46 compounds for the analysis: 16 mono- and sesquiterpenoids, two phenylpropanoids, and an array of di- and triterpenoids, furans, fatty acids, persins, and other metabolites (Table 1). Most of these metabolites were present in an analysis made in other Hass avocado orchards by García-Rodríguez et al. (2016), which includes the list of metabolites and their Kovats indices.

The peaks obtained by GC-FID were matched with those obtained by GC-MS, using the retention times relative to tetradecane obtained in both chromatographs.

### Relating phytochemical diversity metrics with plant enemies

To determine if phytochemical diversity at the scale of individual trees and orchards was related to the incidence of the studied plant enemies, we obtained several metrics. At the tree scale, aside from grouping the trees under chemotypes, we calculated the alpha diversity (Shannon–Weaver diversity index) and beta diversity (defined as the chemical differentiation among individuals) (García-Rodríguez et al., 2012; Wetzel & Whitehead, 2020), for each tree using the concentration of each compound relative to tetradecane in each leaf sample. We used the PAST statistical software to calculate alpha and beta diversity (Hammer, 2019). We also calculated: (a) the total concentration of all PSMs; (b) the concentration of monoterpenoids + sesquiterpenoids + phenylpropanoids; (c) the concentration of persins, and (d) the concentration of furans. We performed Pearson’s correlation analysis with all those metrics and the incidence of plant enemies using the Statistica 6.0 software (StatSoft), Excel, or *R* programming language (R-Core Team, 2018). Due to the uneven occurrence of plant enemies among the orchards (possibly due to recent pesticide application), we only included orchards where at least one sampled tree had a particular plant enemy. This reduced the sample size to 222 trees for branch borer, 223 for PCB, 194 for red mite, 222 for thrips, and 166 for whitefly. The sample size for fruit rot and scab was 236 trees.

**Table 1:** Metabolites found in the foliar hexanic extract of Hass avocado (*Persea americana* cv Hass) analyzed by GC-MS. Concentration is expressed as mg*g^−1^ of dry weight obtained as relative to tetradecane.

RT (min)	Key	Compound^a^	Kri-exp^b^	Kri-theo^c^	Mean ± SE mg g ^−1^
3.88	1	β-pinene	972	974	0.0051 ± 0.0021
3.91	2	β-myrcene	976	988	0.0009 ± 0.0006
4.15	3	α-terpinene	1008	1014	0.0004 ± 0.0002
4.22	4	p-cymene	1017	1020	0.0025 ± 0.0020
4.26	5	limonene	1022	1024	0.0059 ± 0.0030
4.36	6	β-Z-ocimene	1035	1044	0.0009 ± 0.0005
5.3	7	γ-terpinene	1159	1054	0.0020 ± 0.0010
5.48	8	α-terpineol	1083	1186	0.0062 ± 0.0013
5.52	9	estragole	1088	1196	0.0589 ± 0.0134
6.73	10	eugenol	1359	1356	0.0214 ± 0.0043
7.18	11	β-caryophyllene	1427	1417	0.0354 ± 0.0051
7.45	12	α-humulene	1469	1452	0.0214 ± 0.0048
7.55	13	germacrene D	1484	1484	0.0504 ± 0.0081
7.77	14	α-farnesene	1519	1505	0.0134 ± 0.0023
7.82	15	δ-cadinene	1527	1522	0.0340 ± 0.0050
8.07	16	germacrene B	1568	1559	0.0192 ± 0.0043
8.17	17	germacrene-4-ol	1584	1574	0.0574 ± 0.0064
8.2	18	caryophyllene oxide	1589	1582	0.0227 ± 0.0039
9.2	19	myristic acid	1751	1761	0.0267 ± 0.0048
9.51	20	octadecane	1802	1800	0.0438 ± 0.0046
9.63	21	farnesylacetate	1822	1821	0.0537 ± 0.0054
9.96	22	1-hexadecanol	1877	1874	0.1183 ± 0.0081
10.5	23	palmitic acid	1960	1964	0.0263 ± 0.0039
11.1	24	methyl linoleate	2070	2075	0.0416 ± 0.0087
11.3	25	phytol	2112	2122	0.0458 ± 0.0092
11.6	26	ethyl linoleate	2164	2169	0.1176 ± 0.0109
11.9	27	2-[(8Z,11Z)-Heptadeca-8-11-dieneyl]furan	2207	NA	0.0386 ± 0.0092
12.4	28	tricosane	2298	2300	0.0626 ± 0.0113
13.1	29	3-Ethyl-3-hydroxy-5α-androstan-17-one	2435	NA	0.0236 ± 0.0031
13.3	30	17-pentatriacontene	2473	NA	0.0211 ± 0.0021
13.4	31	2-hexadecenoic acid, methyl ester, (E)-	2484	NA	0.0722 ± 0.0088
13.5	32	linoleic acid ethyl ester	2502	NA	0.7205 ± 0.0558
13.5	33	unknown1	2502	NA	0.1079 ± 0.0213
13.5	34	unknown2	2515	NA	0.0364 ± 0.0035
13.6	35	unknown3	2515	NA	0.0224 ± 0.0027
13.9	36	17-pentatriacontene	2531	NA	0.0599 ± 0.0067
14.1	37	β-stigmasterol	2592	NA	0.0674 ± 0.0069
14.1	38	7,10,13-eicosatrienoic acid, methyl ester 1	2633	NA	0.1400 ± 0.0115
14.2	39	7,10,13-eicosatrienoic acid, methyl ester 2	2658	NA	0.0247 ± 0.0022
14.4	40	heptacosane	2696	2700	0.0399 ± 0.0038
14.8	41	persin 1^d^	2766	NA	0.1577 ± 0.0132
14.8	42	persin 2^d^	2774	NA	0.0284 ± 0.0046
15.2	43	squalene 1	2842	2847	0.0957 ± 0.0077
15.2	44	squalene 2	2849	2847	0.0541 ± 0.0076
15.6	45	eicosane	2907	2900	0.0205 ± 0.0032
16.4	46	β-tocopherol	3080	3076	0.1043 ± 0.0135

**Notes.**

a Compounds are listed in order of elution on an HP-5MS column.

RT retention time NA not available

b Kri-exp: Kovats retention index on HP-5MS obtained experimentally.

c Kri-theo: Kovats retention index from literature (see experimental section).

d Identification by comparison with mass fragmentation patterns from literature.

At the orchard scale, we calculated the diversity (Shannon, Simpson, and Evenness) and dominance for each orchard based on the number and abundance of the chemotypes present in it. To determine if the trees grouped at the orchard level were correlated to the average incidence of plant enemies, we calculated the average diversity of trees and the average chemical differentiation of the trees within each orchard to obtain Average Chemical Distance (ACD) and Standard Deviation for Chemical Distance (STD CD). For that, we calculated a distance matrix using city-block (Manhattan) distance with the PAST software. Those metrics were correlated with plant enemy incidence using Statistica 6.0 software. Due to the uneven incidence of plant enemies among the orchards, the sample size was reduced to 16 orchards for branch borer, 16 for PCB, 14 for red mite, 16 for thrips, and 12 for whitefly. The sample size for fruit rot and scab was 17 orchards.

### Classification of trees based on their chemotypes.

Mono- and sesquiterpenoids occur in mixtures where the relative proportion of each compound is under tight genetic control in some trees; a particular compositional pattern of terpenoids is uniform in the mature foliage of an individual (Langenheim, 1994; Sánchez-Hidalgo, Martínez-Ramos & Espinosa-García, 1999; Bustos-Segura et al., 2017).

**Figure 1:**
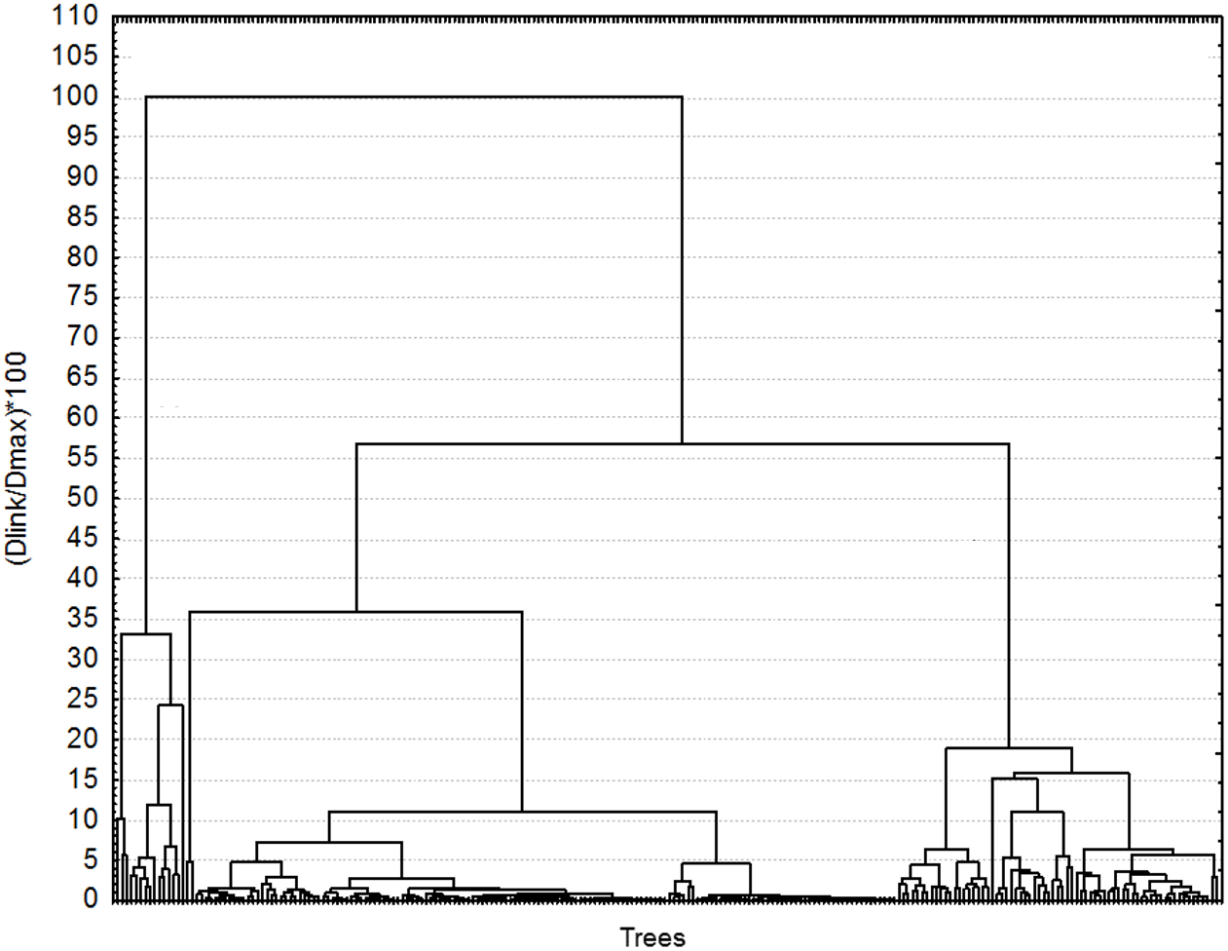
Classification of 236 Hass avocado trees according to their chemotype. Each terminal line represents one tree. All trees that were dissimilar from others by less than 5% were grouped in the same chemotype. Cluster analysis was made using city-block (Manhattan) distances and Ward’s method linkage algorithm.

To classify the trees according to their chemotype, a matrix was built of 236 trees by 18 metabolites (16 mono- and sesquiterpenoids, and two phenylpropanoids), which compositional pattern is probably under tight genetic control (Lavi et al., 1993a; Lavi et al., 1993b; Wu et al., 2007; Gershenzon et al., 2012; Bustos-Segura et al., 2017). The trees in the matrices were classified with hierarchical cluster analysis using Ward’s linkage method and city-block (Manhattan) distances using Statistica 6.0. Trees were grouped in the same chemotype when the dissimilarity within the cluster was equal or smaller than 5% (Fig. 1). The resulting chemotypes were grouped according to their frequency in the sample: high, medium, and low frequency.

### Plant enemies, chemotypes, and leaf metabolites

To determine if the plant enemy species had a differential incidence on the groups of chemotypes they were analyzed with ANOVA using Statistica 6.0 software (Statsoft, Inc). To determine if the trees defined by their chemotypes and the 46 leaf metabolites were related to the plant enemies, we performed Canonical Correspondence Analyses (CCA) using the CCA function in the ‘vegan’ package (Oksanen et al., 2018) from the R software (R-Core Team, 2018). We used the Akaike information criterion to simplify models using the ‘step’ function in the ‘vegan’ package. Afterward, we performed a multivariate analysis of similarity (ANOSIM) (Clarke, 1993; Warton, Wright & Wang, 2012) to test if the trees grouped according to their chemotypes frequencies (high, medium, or low) related differentially to the plant enemies. This method uses a dissimilarity matrix built with the concentration of the leaf’s metabolites. If the statistic obtained, the *R*-value, is zero, then the dissimilarities among and within groups are the same. If the *R*-values are higher than zero, then the dissimilarities within groups are smaller than dissimilarities among them. The CCA analysis detected four groups of trees; to corroborate this observation, the package “Nbclust” was used (Malika et al., 2014). After groups were clearly identified, we tested if they were significantly different with an analysis of similarities (ANOSIM function of vegan package).

## Results

### Testing the more diversity-better defense hypothesis

This hypothesis predicted negative relationships between the metrics of phytochemical diversity and the incidence of plant enemies at the individual and orchard scales. However, the correlations of the incidence of all plant enemies with phytochemical diversity metrics were either not significant or significant with a very low positive *r* (Tables 2 and 3). A correlation matrix between plant enemies and phytochemical metrics at the tree scale (the total concentration of mono- sesquiterpenoids, and phenylpropanoids (MT+ST+PP), the total concentration of furans, the total concentration of persins, and alpha diversity), yielded several significant correlations, few were negative as the hypothesis of more diversity-better defense predicted (Table 2). The red mite regressed negatively with persins; the branch borer, positively with the sum MT+ST+PP and with average chemical distance, but negatively with furans and persins. The whitefly regressed positively with persins. The scab fungus disease showed no significant relationships, and the PCB showed a negative correlation with MT+ST+PP. We found positive, negative, or neutral correlations with different pests and pathogens and the total concentration of bioactive metabolites: MT+ST+PP, furans, and persins. The average chemical diversity and its standard deviation showed only positive correlations with insect pests.

**Table 2:** Pearson’s correlations between phytochemical metrics and the incidence of avocado pests and pathogens at the individual tree scale. Values in bold show significant correlations.

	Red mite	Branch borer	Thrips	Whitefly	Fruit rot	Scab	PCB
Sum MT+ST+PP	*r* = − 0.005	**r = 0.425**	*r* = − 0.121	*r* = − 0.112	**r = −0.13**	*r* = 0.004	**r = −0.146**
	*p* = 0.944	**p < 0.001**	*p* = 0.074	*p* = 0.153	**p = 0.048**	*p* = 0.945	**p = 0.03**
Furans total concentration	*r* = 0.017	*r* = − 0.087	*r* = 0.049	*r* = 0.101	*r* = 0.067	*r* = 0.002	*r* = 0.085
	*p* = 0.816	*p* = 0.198	*p* = 0.468	*p* = 0.197	*p* = 0.309	*p* = 0.98	*p* = 0.21
Persins total concentration	*r* = − 0.091	**r = −0.159**	*r* = 0.127	*r* = 0.144	**r = 0.182**	*r* = − 0.038	*r* = 0.037
	*p* = 0.207	**p = 0.018**	*p* = 0.061	*p* = 0.065	**p = 0.005**	*p* = 0.561	*p* = 0.581
Alpha diversity (H)	*r* = 0.009	*r* = 0.034	*r* = − 0.094	**r = −0.168**	**r = −0.142**	*r* = 0.028	*r* = − 0.082
	*p* = 0.906	*p* = 0.611	*p* = 0.164	**p = 0.031**	**p = 0.03**	*p* = 0.671	*p* = 0.223
Average chemical distance	*r* = 0.023	**r = 0.202**	*r* = 0.073	*r* = 0.09	*r* = − 0.057	*r* = − 0.031	*r* = − 0.103
	*p* = 0.75	**p = 0.003**	*p* = 0.278	*p* = 0.253	*p* = 0.385	*p* = 0.641	*p* = 0.127
STD chemical distance	*r* = 0.08	**r = 0.133**	**r = 0.213**	**r = 0.342**	*r* = 0.005	*r* = − 0.003	**r = −0.163**
	*p* = 0.272	**p = 0.049**	**p = 0.001**	**p < 0.001**	*p* = 0.938	*p* = 0.965	**p = 0.015**

**Notes.**

Significance level: p ≤ 0.029, based on the Benjamini–Hochberg procedure for 42 comparisons. Due to the uneven incidence of plant enemies among the orchards, we only included orchards where at least one sampled tree had a particular plant enemy. This reduced the sample size to 222 trees for branch borer, 223 for PCB, 194 for red mite, 222 for thrips, and 166 for whitefly. The sample size for fruit rot and scab was 236 trees; ns= non-significant.

**Table 3:** Pearson’s correlations of phytochemical diversity metrics and average plant enemy incidence at the orchard scale.

		Red mite			Branch borer			Thrips			Whitefly			Fruit rot			Scab			PCB	
		*r*	*p*		*r*	*p*		*r*	*p*		*r*	*p*		*r*	*p*		*r*	*p*		*r*	*p*
avg Chem. distance		0.044	0.883		0.251	0.349		0.233	0.384		0.299	0.344		0.130	0.620		0.046	0.862		**0.541**	**0.031**
std Chem. Distance		0.035	0.906		0.220	0.413		0.335	0.204		**0.648**	**0.023**		0.030	0.909		0.108	0.681		0.330	0.212
Dominance		0.069	0.816		0.423	0.103		0.017	0.948		0.111	0.731		0.281	0.274		0.052	0.844		0.181	0.503
Simpson		0.069	0.816		0.423	0.103		0.017	0.948		0.111	0.731		0.281	0.274		0.052	0.844		0.181	0.503
Shannon		0.000	0.998		0.396	0.129		0.110	0.685		0.076	0.815		0.335	0.189		0.079	0.765		0.123	0.649
Eveness		0.196	0.501		0.520	0.039		0.197	0.465		0.396	0.202		0.168	0.519		0.060	0.819		0.333	0.207

**Notes.**

STD avg. standard deviation of average

Significance level: *p* < 0.029, based on the Benjamini-Hochberg procedure for 42 comparisons. Due to the uneven incidence of plant enemies among the orchards, the sample size was reduced to 16 orchards for branch borer, 16 for PCB, 14 for red mite, 16 for thrips, and 12 for whitefly. The sample size for fruit rot and scab was 17 orchards.

At the orchard scale, all the metrics regressed against the incidence of the plant enemies had a non-significant effect on most plant enemies (Table 3). The correlations of evenness of the average of the chemical diversity of chemotypes in the orchard and the PCB incidence did not pass the Benjamini–Hochberg test of false discovery rate.

**Figure 2:**
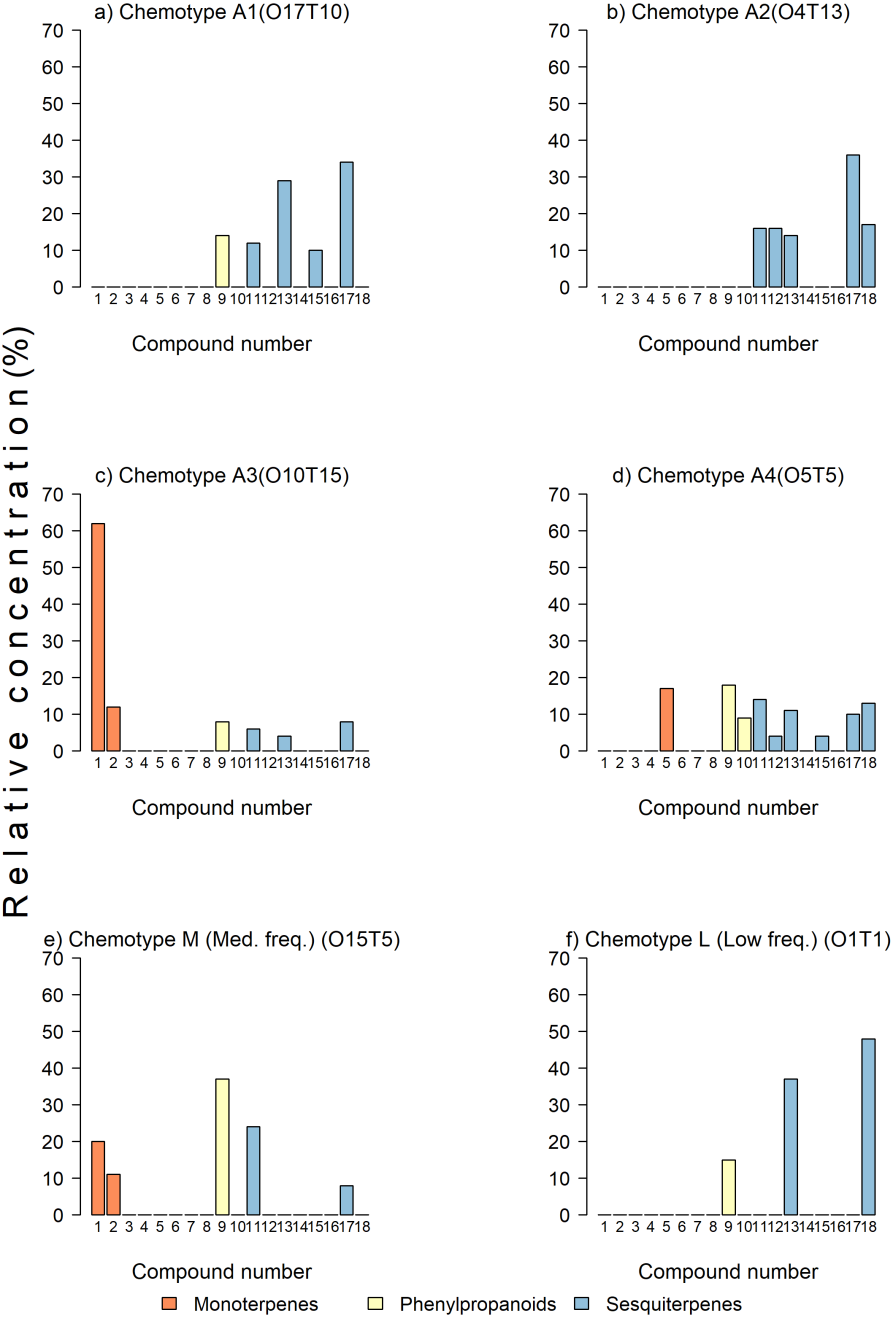
Examples of chemotypes of various abundances in the sample. (A, B, C, D) Trees with high-frequency chemotypes, (E) tree with medium- frequency chemotype, and (F) tree with a low-frequency chemotype. The bars represent percent concentrations relative to the total concentration of monoterpenes (in orange), sesquiterpenes (in yellow), and phenylpropanoids (in blue) present in the leaves of avocado. Key: 1 = *β*-pinene, 2 = *β*-myrcene, 3 = *α*-terpinene, 4 = p-cymene, 5 =limonene, 6 = *β*-Z-ocimene, 7 = *γ*-terpinene, 8 = *α*-terpineol, 9 = estragole, 10 = eugenol, 11 = *β*-caryophyllene, 12 = *α*-humulene, 13 = germacrene D, 14 = *α*-farnesene, 15 = *δ* -cadinene, 16 = germacrene B, 17 = germacrene-4-ol, 18 = caryophyllene oxide.

### Classification of trees in chemotypes

For chemotypes, the cluster analysis grouped the trees in two clusters with a 100% dissimilarity, with most trees remaining in group B in two branches (ca. 55% dissimilarity) (Fig. 1). The trees were grouped in chemotypes when the dissimilitude among them was equal or less than 5%, thus defining 23 chemotypes. There were three to six per orchard (6.35 chemotypes on average). Four chemotypes were the most frequent grouping 70.9% (166) of the trees (abundant chemotype 1 (22), abundant chemotype 2 (24), abundant chemotype 3 (46), and abundant chemotype 4 (74); six chemotypes had medium frequency grouping 20.1% (47) of the trees; the remaining 9% of the trees (23) sampled had low-frequency chemotypes that grouped one to three trees. The highly frequent, medium, and low frequency chemotypes show contrasting chemical composition (Fig. 2). For example, in the abundant chemotypes 1 and 2, the monoterpenes were absent, but the sesquiterpenes predominated: β-caryophyllene, germacrene D, δ-cadinene and germacrene-D-4-ol in A1 and β-caryophyllene, α-humulene, germacrene-D, germacrene-D-4-ol. Also, caryophyllene oxide was predominant in the abundant chemotype 2. In the other two abundant chemotypes, monoterpenes were present: β-pinene was dominant in the abundant chemotype 3, with limonene representing ca. 16% of the mixture. In the tree O15T5, classified as medium-frequency chemotype, the phenylpropanoid estragole predominated representing ca. 38% of the mixture, whereas in the tree O1T1 with a low-frequency chemotype, predominated caryophyllene oxide (47.6%) and germacrene D (37.3%) and estragole (15.1%) with no other compound detected in the mixture.

### Relationship between the frequency of chemotypes and the incidence of pests and pathogens

#### Comparisons of the incidence of plant enemies on chemotypes by ANOVA

**Figure 3:**
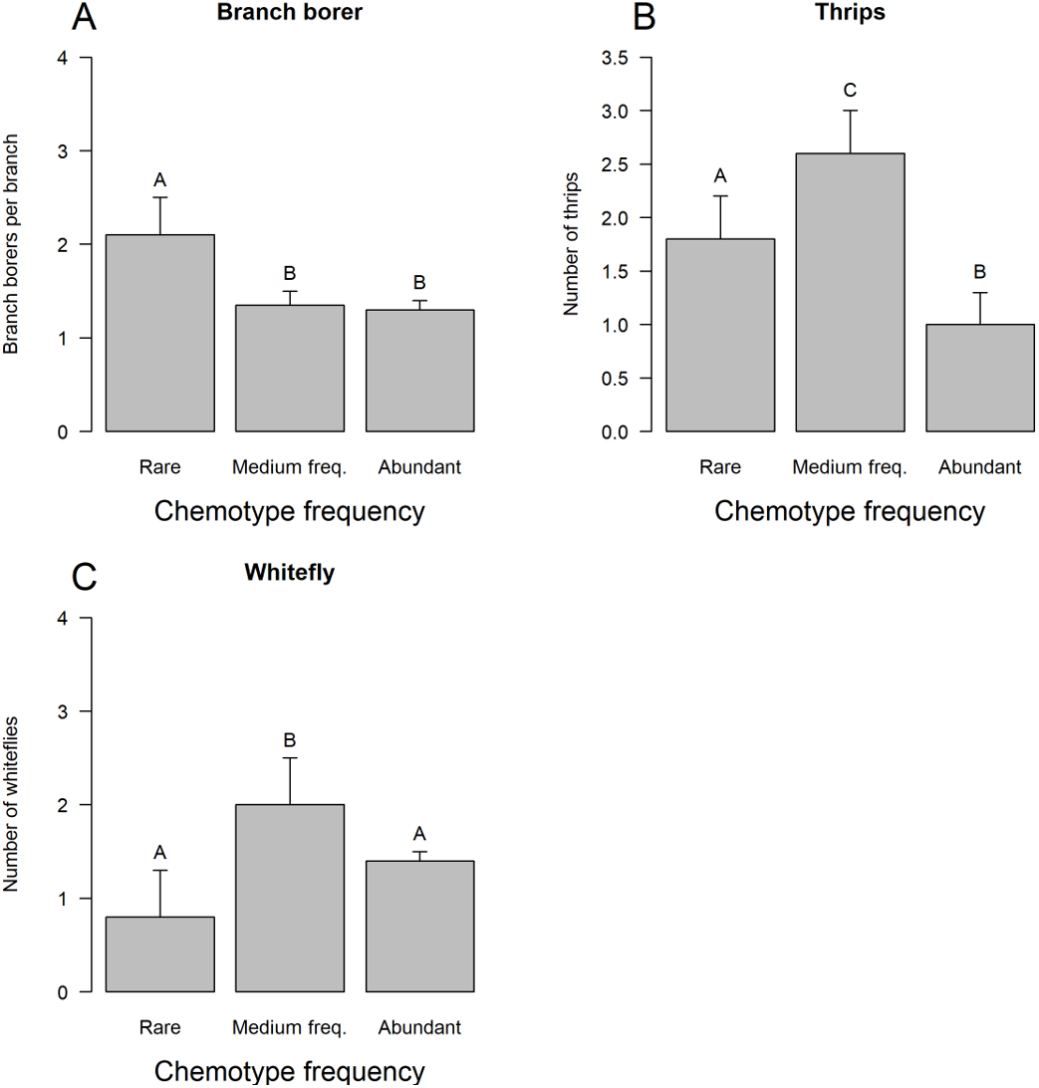
Incidence of plant enemies on tree chemotypes grouped by abundance. LS means, current effects: (A) Branch borer (*F*_(2,217)_ = 43.525, *p* < 0.00001. (B) Thrips (*F*_(2.217)_ = 50.532, *p* < 0.00001). (C) Whitefly (*F*_(2.217)_ = 45.161, *p* < 0.00001). Vertical bars denote 0.95 confidence intervals. Bars with different letters are significantly different.

**Figure 4:**
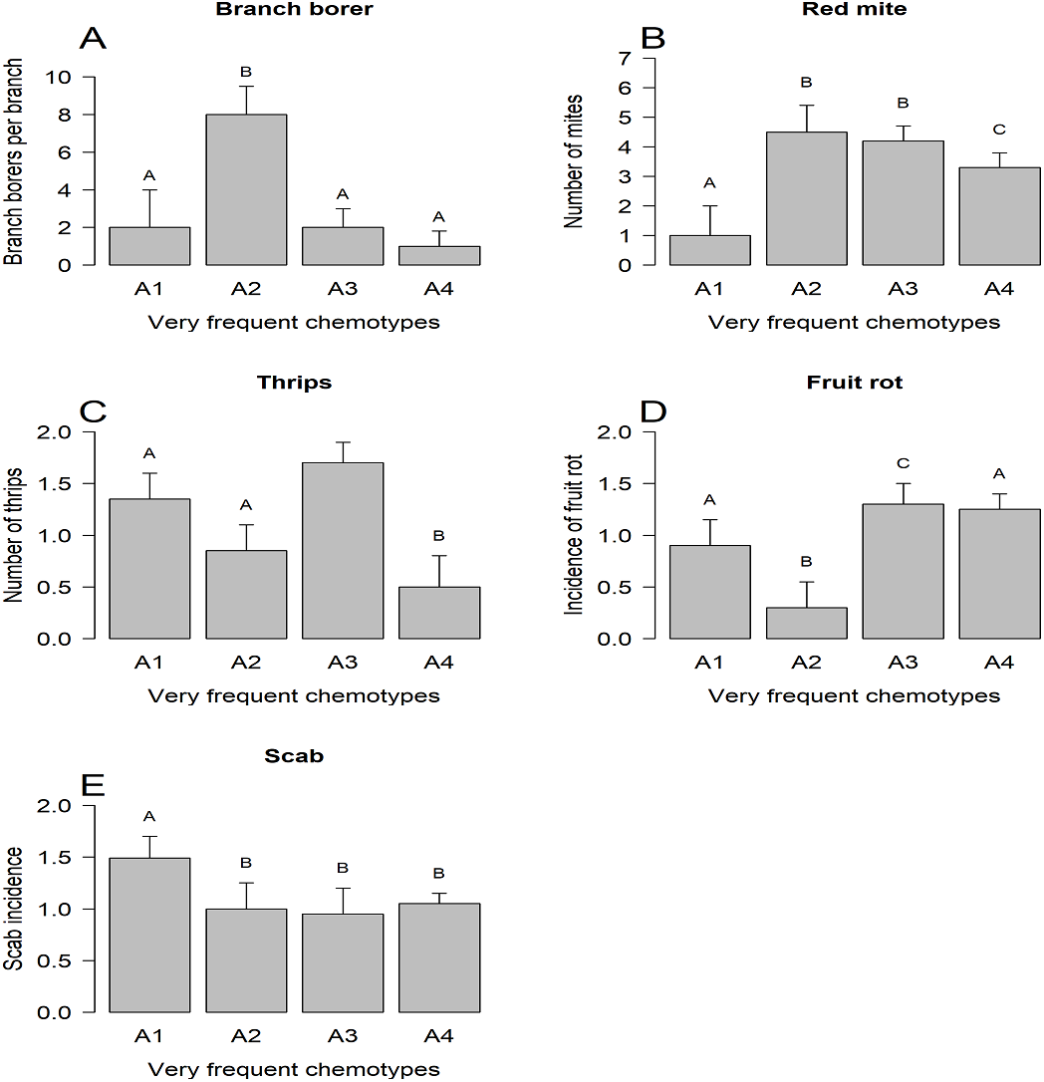
Incidence of plant enemies on trees with high-frequency chemotypes. LS means, current effects: (A) Branch borer (*F*_(3.148)_ = 29.613, *p* < 0.00001). (B) Red mite (*F*_(3.152)_ = 15.895, *p* < 0.00001). (C) Thrips (*F*_(23,148)_ = 17.998, *p* < 0.00001). (D) Fruit rot (*F*_(3,162)_ = 20.966, *p* < 0.00001). (E) Scab (*F*_(3,162)_=5.039, *p* = 0.0023). Vertical bars denote 0.95 confidence intervals. Bars with different letters are significantly different.

The three groups of chemotypes defined by their frequency associated differentially only with branch borer (Fig. 3A), thrips (Fig. 3B), and whitefly (Fig. 3C): low-frequency chemotypes had a higher load of branch borers than the other two categories, but these chemotypes had the lowest whitefly load. The medium frequency chemotypes had the highest thrips and whitefly loads than the low-frequency and very frequent chemotypes. The group of very frequent chemotypes had trees grouped under four predominant chemotypes (A1 to A4) that displayed differential incidence of branch borer (Fig. 4A), red mite (Fig. 4B), thrips (Fig. 4C), fruit rot (Fig. 4D), and scab (Fig. 4E); whitefly and PCB showed no differential incidence on trees. The trees with the abundant chemotype 2 had the highest load of branch borer but the lowest one of fruit rot. Similarly, trees with the abundant chemotype 1 had the lowest incidence of red mite but the highest scab incidence.

#### Incidence of plant enemies on the trees defined by their chemotypes and single leaf metabolites analyzed by CCA and ANOSIM

**Figure 5:**
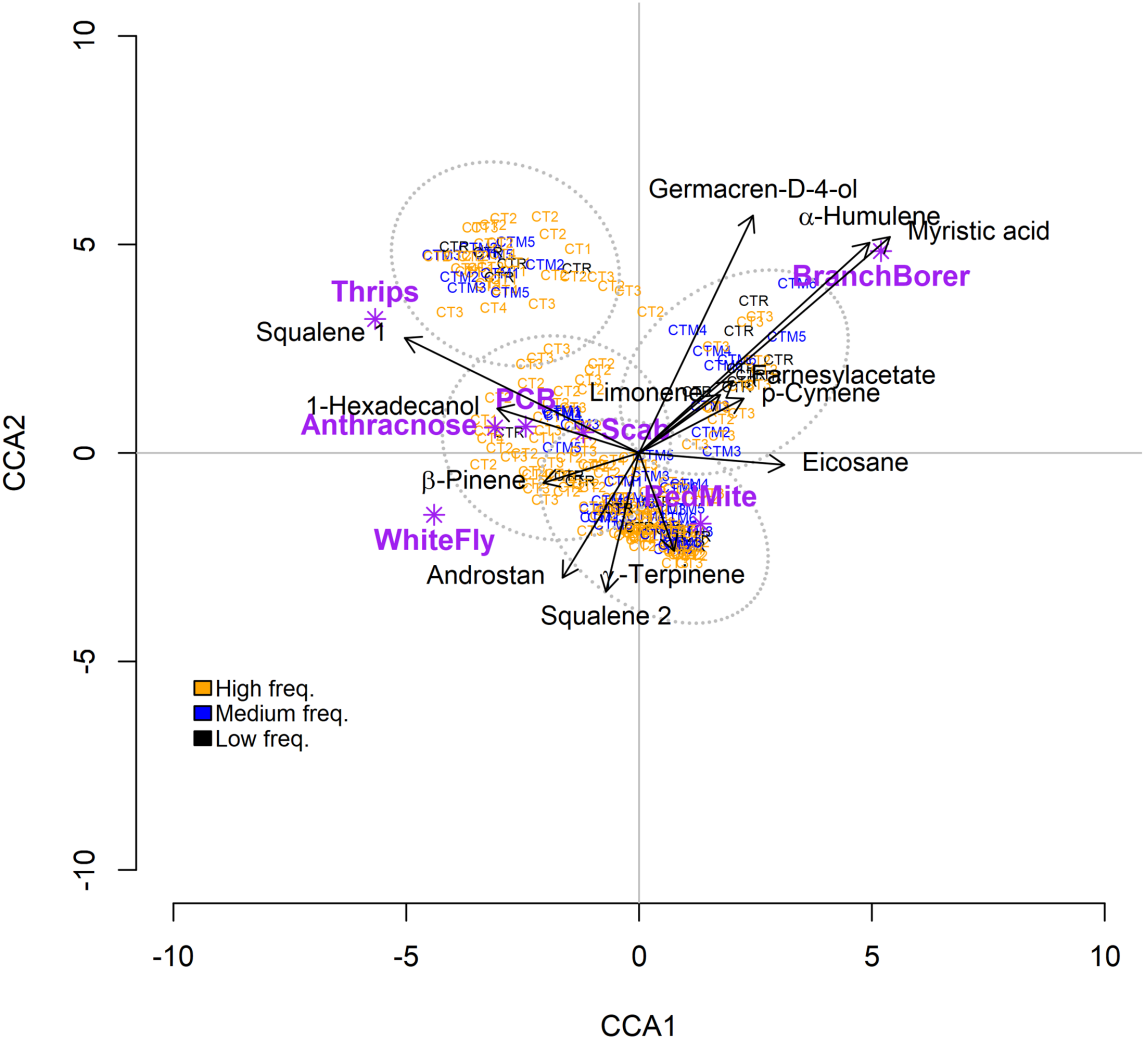
Results of the Canonical Correspondence Analysis (CCA) relating Hass avocado foliar PSMs with the incidence of plant enemies on trees classified by their chemotypes (CT). Results of the Canonical Correspondence Analysis (CCA) relating Hass avocado foliar PSMs with the incidence of plant enemies on trees classified by their chemotypes (CT). The color for the chemotypes label corresponds to their frequency in the 236 trees sample. Orange, high-frequency chemotype; blue, medium-frequency CT; black, low-frequency CT. Androstan =3-Ethyl-3-hydroxy-5α-androstan-17-one.

The CCA ordinated four groups of trees according to their foliar PSMs (but not the frequency of their chemotypes) and plant enemies (Fig. 5). The similarities within groups are significantly higher than similarities among groups (*R*= 0.017; *p* = 0.002). The CCA model is significant but accounting for a low amount of variance (18.6%) (Table 4). Permutation analysis showed that 13 PSMs contributed to the CCA model, 11 of them with a significant influence on plant enemy community structure (Table 4B). Trees with high amounts of myristic acid, α-humulene, and germacrene-D-4-ol are associated with the incidence of the branch borer; thrips are associated with a high concentration of squalene-1. The rest of the plant enemies showed a weak association with several PSMs (Fig. 5). The ANOSIM showed that the trees grouped by the frequency of their chemotypes are not different among them in terms of the incidence of plant enemies (ANOSIM statistic *R*: 0.049 Significance: *p* = 0.064).

**Table 4:** Results of the Canonical Correspondence Analysis for the 46 foliar metabolites in avocado trees and the incidence of plant enemies. a) Influences of metabolites in the foliar compounds on seven pests and pathogens in avocado trees. b) Global significance of the CCA.

**a)**				
**Metabolite**	**Df**	**Chi-Square**	***F***	***Pr(>F)***
β-Pinene	1	0.01027	2.9955	0.017^*^
*p*-Cymene	1	0.00624	1.8197	0.124
Limonene	1	0.00983	2.8696	0.027^*^
γ-Terpinene	1	0.00588	1.7166	0.127
α-Humulene	1	0.04263	12.4406	0.001^***^
Germacren-D-4-ol	1	0.01425	4.1587	0.004^**^
Myristic acid	1	0.01236	3.6061	0.004^**^
Farnesylacetate	1	0.00241	0.7028	0.585
1-Hexadecanol	1	0.01185	3.4572	0.012^*^
3-Ethyl-3-hydroxy-5α-androstan-17-one	1	0.00778	2.2697	0.05^*^
Squalene 1	1	0.03614	10.5466	0.001^***^
Squalene 2	1	0.01091	3.185	0.015^*^
Eicosane	1	0.01511	4.4081	0.004^**^
Residual	220	0.75393		

**b)**		
**Partitioning of scaled Chi-square:**		
	Inertia	Proportion
Total	0.9396	1
Constrained	0.1857	0.1976
Unconstrained	0.7539	0.8024

**Significance test of the constrained variance (0.1857):**	Df	ChiSquare	F	Pr (>F)
Model	13	0.18566	4.1674	0.001^***^
Residual	220	0.75393		

**Notes.**

* *p* < 0.05.

** *p* < 0.01.

*** *p* < 0.001.

## Discussion

We tested several metrics of phytochemical diversity at the scales of individual trees and orchards, but we did not find the expected generalized negative correlation between the incidence of pests and pathogens and phytochemical diversity. The variable richness of foliar PSMs in the chemical phenotypes of the trees did not show any correlation with the incidence of plant enemies. Even with the 23 chemotypes detected, the negative correlation was absent. However, we found some chemotypes associated with differential susceptibility to plant enemies, related either to the composition of the chemotype or to its frequency in the orchards analyzed.

The almost absent negative correlations between phytochemical diversity and plant enemies may be due to several causes, starting with the suitability of our study system to test the more diversity-better defense hypothesis: the amount of variability in a clonal-propagated crop and the widespread pesticide use in the studied region. For the first cause, it could be that the Hass avocado crop has not had time to generate and keep enough genetic variation to enable phytochemical diversity function as a defensive trait. Another possibility to explain the absence of those negative correlations could be that we could not detect a small but significant effect of phytochemical diversity on avocado enemies. A meta-analysis on the consequences of intraspecific adaptive genetic diversity (measured as clone richness among other measures) showed small significant positive effect on productivity at the community and ecosystem scales (Whitlock, 2014). However, genotypic diversity in some crops may have an inconsistent effect on the incidence of herbivores (Tooker & Frank, 2012; Grettenberger & Tooker, 2020).

The recent origin of the avocado Hass variety (Bost, Smith & Crane, 2013) and the common practice of its clonal propagation by grafting, suggested that the studied orchards would show limited variation. However, we found 23 chemotypes throughout the sampled plots, with an average of 6 (3–11) chemotypes per orchard. Thus, despite the clonal origin and propagation of Hass avocado, this crop displays more genetic variation measured in chemotypes than that expected in a cultivar obtained in the last century from a single tree. That may be accounted for by the occurrence of processes as diverse as somatic mutation events, transposons, epigenetic variation, and/or epistasis in the branches of the avocado trees selected by growers in the propagation nurseries (Edwards et al., 1990; Szymkowiak & Sussex, 1996; García-Rodríguez et al., 2016). Although the rate at which somatic mutations appear in avocado trees is unknown, this rate may be high in other clonal plants (Silander, 1985). Additionally, the observed variation may be associated with the appearance of mutants that are resistant to herbivores (Edwards et al., 1990; Ohmart & Edwards, 1991) that would be readily selected by avocado propagators.

Despite that 23 chemotypes were detected in 236 trees, 70.3% of the trees were associated with four predominant chemotypes, which suggests that, at a regional scale, the genetic variation of avocado measured in chemotypes is not very high. A study of the foliar chemical variability of the wild or perhaps semidomesticated *Persea americana* var. *drymifolia* (one of the parental botanical varieties of the Hass avocado) found 47 chemical phenotypes represented in 291 trees from 39 accessions, planted in a germplasm bank (Torres-Gurrola, Montes-Hernández & Espinosa-García, 2009). Thus, the spectrum of Hass avocado chemical diversity might not have been as wide as needed to test effectively the more diversity-better defense hypothesis.

Another possible cause for the absence of negative correlations between phytochemical diversity metrics and plant enemy incidence could be the multiple effects that PSMs may have on plant enemies: a single metabolite or mixture may have stimulant effects on one species, whereas it may have antagonistic effects on another (Langenheim, 1994; Glassmire et al., 2016; Gaillard et al., 2018). That pattern of differential activity of PSM on plant enemies is present in maize and its wild ancestors, showing that their PSMs exert strong antagonism against insect generalists and lowered deterrence or stimulating effects on insect specialists (Gaillard et al., 2018). In contrast to the possible effect of single metabolites, when the PSMs are considered as a set, or as the total concentration of groups of PSMs (Table 2), our results suggest that different sets of foliar metabolites affect the incidence of pests and pathogens on avocado trees. For example, the branch borer correlated positively with the concentration of monoterpenoids, sesquiterpenoids, and phenylpropanoids, but it correlated negatively with the concentration of furans and persins. In contrast, the PCB correlated negatively with the terpenoids and phenylpropanoids that were associated with the branch borer (Table 2). The canonical correspondence analysis (CCA) model correlating the 46 foliar metabolites of the chemical phenotypes and the plant enemy community structure, included 13 metabolites that accounted for 18.57 % of the variation in the incidence of plant enemies. This rather low accounted variability may be due to the differential susceptibility of the plant enemies to the chemicals, yet, the CCA shows clusters of trees with particular chemical compositions associated with certain pests and pathogens (Fig. 5). For example, increased amounts of germacrene-D-ol, α-humulene, and myristic acid correlated with the incidence of the branch borer. However, β-pinene, squalene 2, and androstane- correlated negatively with the branch borer whereas the whitefly shows the opposite pattern (Fig. 5).

The differential effects of various PSMs sets against plant enemies suggest that the sole increase of single PSMs does not necessarily increase the efficacy of plant defense. The PSMs multifunctionality, their activity as components of mixtures, and the differential effects of sets of PSM on plant enemies (Langenheim, 1994; Richards et al., 2016) possibly imply that the phytochemical diversity has a ceiling determined by the cluster of plant enemies attacking a plant population.

The interactions between plants and their enemies may imply polymorphic plant populations in their resistance/susceptibility traits to plant enemies not only in the number of variants but in the frequency of them (Sturgeon, 1979). Our results suggest that trees with the predominant chemotypes have differential susceptibility to the attack of arthropod pests such as branch borer, red mite, and thrips, and the fungus diseases fruit rot and scab (Fig. 4). In contrast, no differential susceptibility for trees of predominant chemotypes was observed for whitefly and PCB. Thus, the incidence patterns of the plant enemies on the chemotypes are heterogeneous: some plant enemies are sensitive to the variation represented by chemotypes, whereas other enemies are insensitive to that phytochemical variation. For example, the trees with the abundant chemotype 2 had the highest incidence of branch borer and red mite, but the lowest one for fruit rot (Fig. 4). The finding of chemotypes associated with differential susceptibility to plant enemies is consistent with different clusters of herbivores attacking different clones of a population, like in the case of goldenrod (*Solidago altissima)* (Maddox & Root, 1987), or the differential susceptibility of *Melaleuca alternifolia* chemotypes to insects and a fungal pathogen (Bustos-Segura, Külheim & Foley, 2015).

The level of resistance to pests and pathogens of plant populations is related to their heterogeneity (reviewed in Tooker & Frank, 2012). However, the degree of phytochemical homogeneity or heterogeneity of the host for a plant enemy depends on the detoxification abilities of the particular pest or pathogen. For example, the sensitivity to heterogeneity in plant resistance for a generalist pathogen, such as *Phytophthora cinnamomi* that can attack plants belonging to over 40 families, will be different from that for a specialist, such as the Abert squirrel that feeds on some chemotypes of a pine species, leaving others untouched (Snyder, 1992). Thus, the increase in Hass avocado chemotypes does not necessarily mean better defense; the new variants should be resistant to at least a plant enemy that may damage the plant population. We showed that the Hass avocado crop is heterogeneous in terms of the incidence of some of its plant enemies. The differential incidence of enemies on trees depending on their chemotypes can be partly associated with their foliar PSMs: this was evident in the CCA analysis where the model that accounted for 18.6 % of the variation of the incidence of plant enemies included 13 PSMs, six of them mono- and sesquiterpenoids (Table 4B). However, most of the differential susceptibility of chemotypes could be due to other traits that co-vary with the chemotypes, and each variant should be evaluated to determine if it contributes to the resistance of the crop to the plant enemies. Therefore, an orchard planted with trees with several chemotypes, with tested differential resistance to the local array of pests and pathogens, will become a viable orchard that will not be affected severely by a single plant enemy.

Our results suggest that phytochemical diversity function as a defensive trait relies more on differentiation among individuals in a population (Massad et al., 2017) than on the sole increase of chemical diversity (Moore et al., 2014). We hypothesize that the relationship between phytochemical diversity and defense against plant enemies is non-linear. There must be a short interval in which increasing the active phytochemical diversity means better defense. Then, the number of variants would reach an upper limit and possibly, a dynamic equilibrium determined by the community of plant enemies. In this upper limit, the number of phytochemical variants and their chemical configuration would determine the structure of the plant enemy community at the same time that phytochemical diversity gets determined by the plant enemies.

## Conclusions

• The hypothesis of more diversity-better defense was not supported by the correlations between the phytochemical diversity and the incidence of pests and pathogens in Hass avocado orchards; this was probably due to a narrow spectrum of phytochemical variability in the crop.
• Despite the clonal origin and propagation of Hass avocado, this crop displays more variation (23 chemotypes) than that expected in a cultivar obtained in the last century from a single tree.
• The type and frequency of the chemotypes were associated with the differential incidence of pests and pathogens. For example, the trees expressing the predominant chemotypes were less damaged by the branch borer, relative to the trees with low or medium frequency chemotypes.
• Our results suggest that phytochemical diversity function as a defensive trait relies more on differentiation among individuals in a population than on the indefinite increase of chemical diversity.
• The differential incidence of pests and pathogens on avocado trees with chemotypes defined by chemotypes of mono- sesquiterpenes, and phenylpropanoids, imply that chemotypes represent potentially useful markers for Hass avocado orchard pest management.

## Supplemental Information

10.7717/peerj.11796/supp-1 Supplemental Information 1 Location of the sampled orchards and number of chemotypes per orchard The total number of chemotypes found in the 236 trees sample was 23 (range 3 to 11 per orchard); masl = meters above the sea level.

10.7717/peerj.11796/supp-2 Supplemental Information 2 Dataset: 46 foliar secondary metabolite content in 236 Hass avocado trees and the incidence of seven plant enemies on those trees This also includes phytochemical diversity metrics at the tree and orchard scale and the chemotype for each tree.
The trees are organized by the orchard. The matrix with those data was used to calculate the phytochemical diversity matrix, to analyze the data with cluster analysis to identify compositional types, and to relate the foliar metabolites with plant enemies using canonical correspondence analysis.

## Additional Information and Declarations

### Field Study Permissions

The following information was supplied relating to field study approvals (i.e., approving body and any reference numbers): This study did not require any permission of authorities or institutions, as the researchers’ activity and leaves recollection were innocuous. Thus, the permission of whoever was taking care of an orchard was enough to enter the orchard and recollect avocado leaves and data on plant enemies. The orchards’ names and locations are included in Table 1.

### Data Availability

The following information was supplied regarding data availability: The concentrations of the foliar secondary metabolites and the incidence of plant enemies for each tree are available in the Supplementary File.
