# Supplementary material for: Implications of the foliar phytochemical diversity of the avocado crop Persea americana cv. Hass in its susceptibility to pests and pathogens
Source: PeerJ. 2021 Jul 20;9:e11796. doi: 10.7717/peerj.11796 (PMC8759378; doi:10.7717/peerj.11796)
Supplement: Supplemental Information 1 — The total number of chemotypes found in the 236 trees sample was 23 (range 3 to 11 per orchard); masl = meters above the sea level. [file peerj-09-11796-s001.docx]

Appendix 1. Location of the sampled orchards and number of chemotypes per orchard. The total number of chemotypes found in the 236 trees sample was 23 (range 3 to 11 per orchard); masl= meters above the sea level.

| Orchard  key | Orchard name and location | Number of sampled trees per orchard | Number of compositional types per orchard |
| --- | --- | --- | --- |
| O1 | “El Tarascón”. 19°28’00”N 101°40’27”W Highway Uruapan –Morelia, detour-Jujucato- Huertas Km 2. 1900 masl. | 15 | 11 |
| O2 | “La Mesa”. 19°14’20”N 101°27’30”W Road Tacámbaro-Pátzcuaro Km 2. 1616 masl. | 16 | 6 |
| O3 | “Plancarte”. 19°26’00”N 101°55’25”W  Road Ziracuaretiro-San Ángel Zurumucapio  Km 5. 1700 masl. | 13 | 6 |
| O4 | “Méndez 1”. 19°25’12.01”N 101°53’57.83”W Road Ziracuaretiro-San Ángel Zurumucapio  Km 1. 1700 masl. | 14 | 8 |
| O5 | “Méndez 2”. 19°26’00”N 101°55’25”W  Road Ziracuaretiro-San Ángel Zurumucapio Km 1. 1700 masl | 14 | 5 |
| O6 | “Medina”. 19°32’25”N 102°21’35”W  Road Tancítaro–Uruapan Km 12. 2264 masl | 12 | 5 |
| O7 | “Villa”. 19°26’39.59”N 101°52’20.95”W  Free road Uruapan- Pátzcuaro  Km 18. 1980 masl | 14 | 5 |
| O8 | “Gaspar”. 19°30’10”N 101°51’15”W  Free road Uruapan-Pátzcuaro Km 21. 1980 masl | 12 | 5 |
| O9 | “El Tarascón 1”. 19°28’00”N 101°40’27”W Highway Uruapan-Morelia, detour Jujucato-Huertas Km 2. 1900 masl | 15 | 9 |
| O10 | “El Tarascón 2”. 19°28’00”N 101°40’27”W Highway Uruapan-Morelia, detour Jujucato-Huertas Km 2. 1900 masl | 13 | 3 |
| O11 | “Gutiérrez”. 19°14’00”N 101°27’30”W  Road Tacámbaro-Pátzcuaro  Km 6. 1616 masl | 14 | 8 |
| O12 | “Torres”. 19°14’00”N 101°27’30”W  Road Tacámbaro-Pátzcuaro  Km 6. 1616 masl | 14 | 3 |
| O13 | “Rancho Las Mesas”. 19°25’18”N 102°07’50”W Road Nuevo Parangaricutiro-Uruapan Km 5. 1880 | 14 | 6 |
| O14 | “Campos”. 19°31’25”N 102°25’00”W  Road Peribán-Uruapan Km 13. 1500 masl. | 15 | 7 |
| O15 | “Sánchez”. 19°31’25”N 102°25’00”W  Road Peribán-Uruapan Km 16. 1500 masl. | 14 | 9 |
| O16 | “Rancho el Copal”. 19°26’00”N 101°55°25”W Road Ziracuaretiro-San Ángel Zurumucapio Km 5. 1700 masl | 13 | 6 |
| O17 | “Sánchez”. 19°25’17”N 101°53’59”W Road Ziracuaretiro-San Ángel  Zurumucapio Km 1. 1400 masl | 14 | 6 |
| Average |  | --- | 6.35 |
